# Quantile Treatment Effect of Zinc Lozenges on Common Cold Duration: A Novel Approach to Analyze the Effect of Treatment on Illness Duration

**DOI:** 10.3389/fphar.2022.817522

**Published:** 2022-02-01

**Authors:** Harri Hemilä, Elizabeth Chalker, Janne Tukiainen

**Affiliations:** ^1^ Department of Public Health, University of Helsinki, Helsinki, Finland; ^2^ Biological Data Science Institute, Australian National University, Canberra, ACT, Australia; ^3^ Department of Economics, University of Turku, Turku, Finland

**Keywords:** anti-infective agents, data interpretation, outcome assessment, quantile regression, statistics, subgroups, treatment heterogeneity, treatment outcome

## Abstract

Calculation of the difference of means is the most common approach when analyzing treatment effects on continuous outcomes. Nevertheless, it is possible that the treatment has a different effect on patients who have a lower value of the outcome compared with patients who have a greater value of the outcome. The estimation of quantile treatment effects (QTEs) allows the analysis of treatment effects over the entire distribution of a continuous outcome, such as the duration of illness or the duration of hospital stay. Furthermore, most of these outcomes have asymmetric distributions with fat tails, and censored observations are not uncommon. These features can be accounted for in the analysis of the QTE. In this paper, we use the QTE approach to analyze the effect of zinc lozenges on common cold duration. We use the data set of the Mossad (1996) trial with zinc gluconate lozenges, and three data sets of trials with zinc acetate lozenges. In the Mossad (1996) trial, zinc gluconate lozenges shortened common cold duration on average by 4.0 days (95% CI 2.3–5.7 days). However, the QTE analysis indicates that 15- to 17-day colds were shortened by 8 days, and 2-day colds by just 1 day, for the group taking zinc lozenges. Thus, the overall 4.0-day average effect of zinc gluconate lozenges in the Mossad (1996) trial is inconsistent with our QTE findings for both short and long colds. Similar results were found in our QTE analysis of the pooled data sets of the three zinc acetate lozenge trials. The average effect of 2.7 days (95% CI 1.8–3.3 days) was inconsistent with the effects on short and long colds. The QTE approach may have broad usefulness for examining treatment effects on the duration of illness and hospital stay, and on other similar outcomes.

## Introduction

Evaluation of the effects of a particular drug or other medical intervention should not focus only on the average effect. There may be heterogeneity in the effect on an outcome not only with respect to some baseline variables, but also with respect to the outcome itself.

Analysis of quantile treatment effects (QTEs) enables examination of treatment effects over the entire distribution of a continuous outcome such as the duration of illness or the duration of hospital stay (Koenker, 2005; Frölich and Melly, 2010; Koenker, 2017; Koenker et al., 2017). It allows separate analyses of effects on short and long durations of illness, and thereby it is useful in the analysis of potential heterogeneity in the treatment effect. Furthermore, it takes into account outliers or censored observations at the long-duration tail. QTEs have been increasingly analyzed in econometrics (Buchinsky, 1994; Schiele and Schmitz, 2016; Ohrnberger et al., 2020), and their use in medicine has also been encouraged (Lê Cook and Manning, 2013; Hong et al., 2019; Staffa et al., 2019; Yazdani et al., 2021). In practice, QTEs can be estimated with quantile regression (Koenker, 2005; Frölich and Melly, 2010; Koenker, 2017; Koenker et al., 2017), and implemented with standard statistical software (e.g., R *quantreg* and Stata *qreg* packages).

In this study, we used the QTE approach to analyze the effect of zinc lozenges on common cold duration. In 1984, Eby et al. published the results of a randomized controlled trial (RCT) showing that zinc gluconate lozenges increased the rate of recovery from the common cold, but a substantial proportion of patients had censored observations (Eby et al., 1984). In another RCT, Mossad et al. (1996) found that zinc gluconate lozenges reduced the duration of colds on average by 4.0 days (Hemilä, 2017). Finally, a meta-analysis of three RCTs on zinc acetate lozenges (Petrus et al., 1998; Prasad et al., 2000; Prasad et al., 2008) estimated that the duration of colds was reduced on average by 2.7 days (Hemilä et al., 2016). The individual patient data were available for the above studies, allowing the current QTE analysis to examine the effects of zinc lozenges over the entire distribution of common cold duration.

## Methods

### Data of the Included Trials

Mossad et al. (1996) published their findings as survival curves. The numbers of common cold patients recovering each day were measured and are available (Hemilä, 2011; Hemilä, 2017). The data sets of the three RCTs on zinc acetate lozenges (Petrus et al., 1998; Prasad et al., 2000; Prasad et al., 2008) were provided by the authors of the trials and were used in the analyses on the average effect on cold duration (Hemilä et al., 2016) and on the recovery rate (Hemilä et al., 2017). In the Mossad trial there were eight censored observations (8% of the total): in the placebo group there were two on day 7, one on day 15, one on day 16, and two on day 19; and in the zinc group there was one on day 9 and one on day 11. There were no censored observations in the zinc acetate lozenge trials.

### Statistical Analysis

We used the *sqreg* command in Stata to construct the QTE estimates and their 95% CIs. For the Mossad trial, we imputed the duration as the day of censoring; four of the censored observations were beyond the 93rd percentile and this imputation has minimal influence on our analysis. The code for the calculation of the QTE analysis of the Mossad trial is shown in the Supplementary file. QTE figures generated with the R packages *quantreg* and *qte* (Koenker, 2021; Callaway, 2019) are shown in the Supplementary Figures S1, S2. Because of the few cases with censored data in the Mossad trial, we also constructed a QTE curve with 95% CIs generated with the R program *crq* (Supplementary Figure S3), which takes into account censored data (Koenker, 2008). The differences between the 95% CIs are not substantial (Figure 2A vs. Supplementary Figure S3).

## Results

The characteristics of the participants and the contexts of the trials are described in the trial reports (Mossad et al., 1996; Petrus et al., 1998; Prasad et al., 2000; Prasad et al., 2008), and were summarized in previous analyses (Hemilä, 2011; Hemilä et al., 2016; Hemilä, 2017; Hemilä et al., 2017). In brief, all trials were randomized and placebo controlled. Mossad et al. (1996) studied employees of the Cleveland Clinic with a mean age of 38 years. Two of the zinc acetate lozenge trials recruited volunteers from Detroit Medical Center with mean ages 35 and 37 years (Prasad et al., 2000; Prasad et al., 2008), and the third recruited volunteers from the University of Texas with a mean age of 26 years (Petrus et al., 1998). Both sexes were equally represented over the trials. All trials recruited patients with natural colds acquired in the community, and the trials tested the treatment effect of zinc lozenges.

**FIGURE 1:**
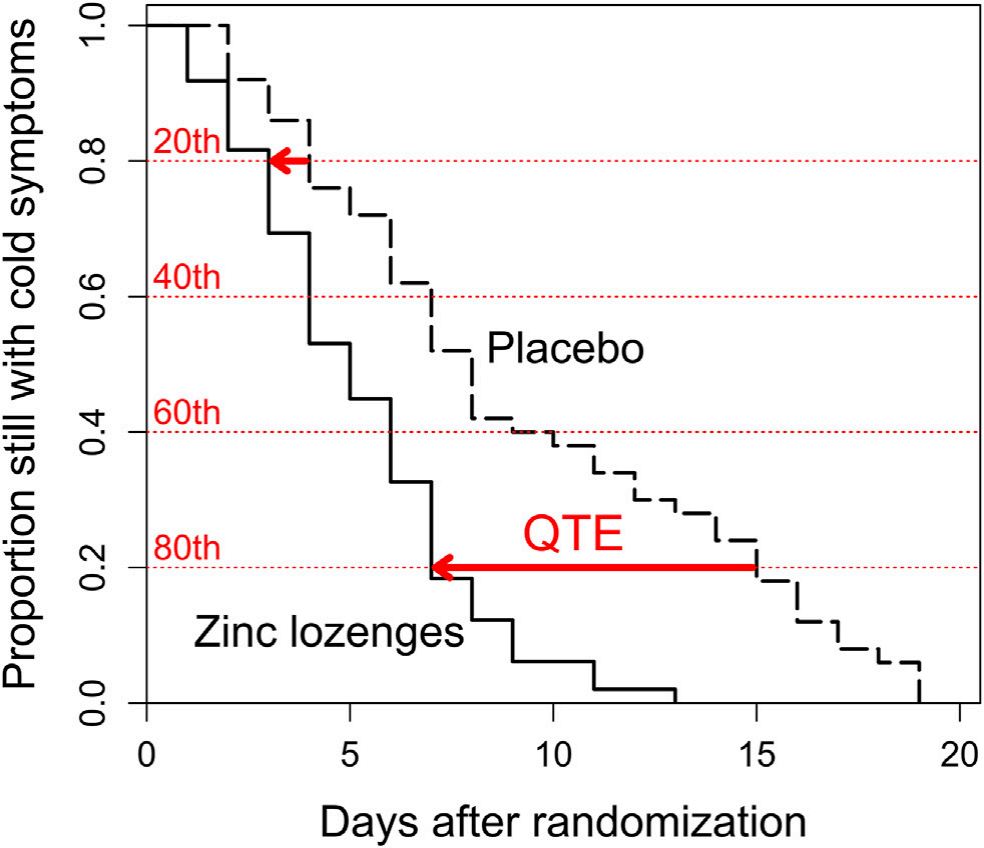
Recovery from the common cold and illustration of the quantile treatment effect (QTE) in the Mossad et al. (1996) trial. The sizes of the steps downwards indicate the number of patients who recovered on a particular day. The red horizontal dotted lines indicate the 20th, 40th, 60th, and 80th percentiles of the distribution of common cold duration, starting with the shortest colds from the top downwards, compare with Figure 2A. The horizontal red arrows indicate the QTE effects at the 20th and 80th percentiles.

The concept of the QTE is illustrated through the survival curves in Figure 1 using the Mossad trial as an example. The horizontal distance between the survival curves of the placebo and zinc groups indicates the QTE. For example, the QTE at the 80th percentile level is shown by the lower red solid arrow. The 80th percentile duration was 15 days in the placebo group and 7 days in the zinc lozenge group. Thereby the QTE at the 80th percentile level is an 8-day reduction in common cold duration for those receiving zinc gluconate lozenge treatment. Similarly, at the 20th percentile, the QTE effect is 1 day, based on 3 days in the zinc group and 4 days in the placebo group, shown by the upper red solid arrow.

**FIGURE 2:**
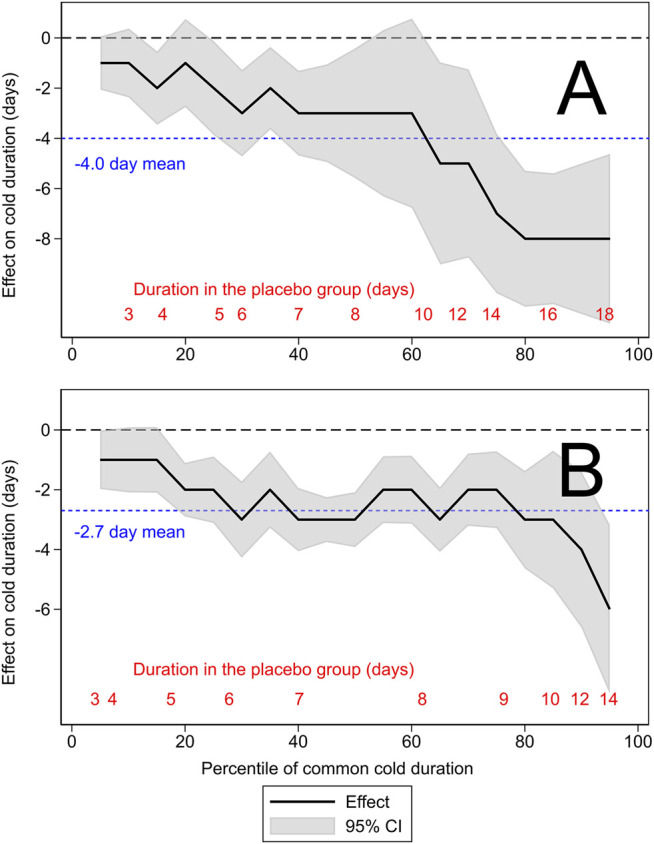
The quantile treatment effect (QTE) of zinc lozenges on common cold duration. **(A)** The Mossad et al. (1996) trial and **(B)** the pooled results of three zinc acetate lozenge trials (Petrus et al., 1998; Prasad et al., 2000; Prasad et al., 2008). The horizontal axis shows the distribution of the duration of colds by percentiles. The continuous black lines indicate the QTE of zinc lozenges and the gray shadow indicates its 95% CI. The horizontal black dashed lines indicate the null effect. The blue dotted line in panel **(A)** shows the previously calculated 4.0-day mean effect in the Mossad trial (Hemilä, 2017), and in panel **(B)** shows the 2.7-day mean effect of zinc acetate lozenges (Hemilä et al., 2016). The red figures at the bottom indicate the lowest percentile level for the indicated common cold duration in the placebo group. For example, in panel **(B)**, the 7-day colds cover the percentile range from 40th to 62nd, which corresponds to 21 patients, as the total number of patients in the placebo groups was 97. The program used for the generation of this figure is shown in the Supplementary Material.

In Figure 2, the distribution of common cold duration in the placebo group is shown on the horizontal axis as percentiles. The difference between the treatment and placebo groups is shown as the QTE on the vertical axis. The continuous black lines indicate the QTE, with the gray shadow indicating its 95% CI. The black dashed lines indicate the null effect level. The blue dotted lines indicate the previously estimated reductions in common cold duration by 4.0 days in the Mossad trial with zinc gluconate lozenges (Figure 2A) and by 2.7 days in the pooled data of the three zinc acetate lozenge trials (Figure 2B).

The QTE of zinc lozenges is heterogeneous on the absolute scale, i.e., in the effect on the duration of illness in days. The average effect of a 4.0-day (95% CI 2.3–5.7 days) reduction in common cold duration in the Mossad trial is seen only in a narrow range around the 60th percentile, corresponding to approximately 10-day colds in the placebo group (Figure 2A). Thus, the uniform 4-day effect is inconsistent with the QTE for shorter and longer colds. The QTE analysis indicates that zinc gluconate lozenges may shorten 15- to 17-day colds by up to 8 days, but the 2-day colds are shortened only by 1 day.

Although the average effect of a 2.7-day (95% CI 1.8–3.3 days) reduction of cold duration in the group receiving zinc acetate lozenges in the pooled data appears reasonable over the range from the 20th to the 80th percentile, corresponding to common cold duration from 5 to 9 days in the placebo group, it exaggerates the effect of zinc acetate lozenges on short colds, and underestimates the effect on long colds (Figure 2B). At both ends of the distribution, the 95% CI range of the QTE is inconsistent with the average 2.7-day effect.

## Discussion

When randomized trials are planned, minimum sample size calculations are carried out to ensure that the trial will have sufficient power to yield meaningful information (Wittes, 2002; Moher et al., 2010). Sample size calculations are usually based on an assumed uniform effect over the participant population, and for continuous variables an effect on the absolute scale (using the units of the measurement in question) is usually assumed (Wittes, 2002). Consequently, the analysis of observations is also based on the assumption of a uniform effect and most medical literature on treatment effects is focused on average effects. However, biology is complex and a single average effect may not apply over all participant subpopulations.

Subgroup analysis by baseline variables is one option to analyze heterogeneity in treatment effects. A zero or even negative overall average effect may hide substantial health benefits for small subgroups for whom receiving the drug may be a matter of life and death. However, many subgroup analyses have been carried out improperly, and therefore the approach has been discouraged (Altman, 1998; Assmann et al., 2000; Freemantle, 2001; Hernández et al., 2006; Moher et al., 2010). Nevertheless, Lagakos commented that “avoiding any presentation of subgroup analysis because of their history of being over-interpreted is a steep price to pay for a problem that can be remedied by more responsible analysis and reporting” (Lagakos, 2006). Cautious subgroup analysis has been encouraged also by other authors (Feinstein, 1998; Rothwell, 2005; Hemilä and Kaprio, 2011).

Quantile regression is a well-established method and widely used in econometrics (Buchinsky, 1994; Koenker, 2005; Frölich and Melly, 2010; Schiele and Schmitz, 2016; Koenker, 2017; Koenker et al., 2017; Ohrnberger et al., 2020), but so far little used in clinical medicine though its use has been recently encouraged (Lê Cook and Manning, 2013; Hong et al., 2019; Staffa et al., 2019; Yazdani et al., 2021). Within this field, the analysis of QTE allows a different approach to examine heterogeneity in treatment effects. Analysis of the QTE is based on the comparison of the distributions of the outcome in the treatment and control groups, assuming that the quantile levels in both distributions correspond to each other (Figure 1). The QTE analysis shows the variation in treatment effect over the distribution of illness duration in the control group (Figure 2). Analysis of heterogeneity with the QTE approach does not require potentially arbitrary choices of variables or binning as does analysis of heterogeneity with respect to baseline variables.

Previous meta-analyses have indicated that properly composed zinc lozenges can shorten common cold duration, while negative findings can be largely explained by shortcomings in the composition of lozenges or in study protocols (Eby, 2001; Eby, 2004; Eby, 2010; Hemilä, 2011; Hemilä et al., 2016; Hemilä et al., 2017; Hemilä et al., 2020). The efficacy of zinc lozenges has been examined on the absolute scale (reduction in days of illness) (Eby, 2004; Eby, 2010; Hemilä et al., 2016), and on the relative scale (percentage reduction and rate ratio of recovery) (Hemilä, 2011; Hemilä et al., 2016; Hemilä et al., 2017; Hemilä et al., 2020). The current absolute-scale QTE analysis indicates that the overall mean effects on reduction in days of illness poorly capture the effect of zinc lozenges (Figure 2). Previous QTE analyses on vitamin C for COVID-19 outpatients (Hemilä et al., 2021), and on nasal carrageenan for common cold patients (Hemilä and Chalker, 2021) also did not find support for a uniform absolute effect. A uniform relative effect may often capture the treatment effect better than a uniform absolute effect (Hemilä, 2017).

In clinical medicine, there is usually greater interest in the effect of a treatment on longer illness duration than shorter. The analysis of QTE yields this information. The 8-day reduction in common cold duration for those using zinc lozenges compared with the placebo-group with 15- to 17-day colds in the Mossad trial is a much more clinically important finding than the 1-day reduction in the 2-day colds (Figure 2A). Such variation in the treatment effect is masked in the calculation of the 4-day overall average effect. Furthermore, subgroup analysis by baseline variables will not reveal such a divergence in the effect on longer and shorter colds.

Many RCTs have such a small sample size that they are only able to answer the question of whether there is evidence of an average effect. In such cases, it may not be possible to undertake an informative QTE analysis. Nevertheless, in some cases QTE analysis can yield useful information from a single RCT (Figure 2A; Hemilä et al., 2021). Furthermore, if there are individual patient data available, a meta-analysis of several trials with the QTE approach can be more informative than just calculating the average effect (Figure 2B; Hemilä and Chalker, 2021).

The QTE approach may have broad usefulness for examining treatment effects on the duration of various illnesses, the duration of hospital stay and ICU stay, and on other similar outcomes. These outcomes usually have asymmetric distributions with fat tails, and censored observations are not uncommon, yet these features can be taken into account with analysis of the QTE.

Using QTEs also allows for conditioning on baseline variables, for example, to increase statistical precision in RCTs or to account for confounders in observational studies, even with censored observations (Koenker, 2008). Implementation of QTE estimation with quantile regression also facilitates studying the effect of heterogeneity in an even more nuanced way by estimating quantile interaction effects between the treatment indicator and the baseline variables.

In conclusion, our study illustrates that the analysis of the QTE can yield useful information about the distribution of treatment effects on common cold duration. The QTE analysis is likely to be useful in the analysis of many clinically relevant continuous outcomes such as the duration of illness, and the duration of ICU stay or hospital stay.

## Data Availability

The original contributions presented in the study are included in the article/Supplementary Material. Further inquiries can be directed to the corresponding author.
