# Supplementary material for: Quantile Treatment Effect of Zinc Lozenges on Common Cold Duration: A Novel Approach to Analyze the Effect of Treatment on Illness Duration
Source: Front Pharmacol. 2022 Feb 1;13:817522. doi: 10.3389/fphar.2022.817522 (PMC8844493; doi:10.3389/fphar.2022.817522)

Quantile treatment effect of zinc lozenges on common cold duration: a novel approach to analyze the effect of treatment on illness duration

Supplementary Material

Harri Hemilä, Elizabeth Chalker, Janne Tukiainen

2021-12-23

Contents **Page**

Data of the analyzed trials: Mossad (1996) 2

Data of the analyzed trials: three Zinc acetate trials 3

STATA *sqreg* procedure for constructing Fig. 2A 4

Figure S1: *quantreg* package of R: *rq* procedure 5

Figure S2: *qte* package of R: *ci.qte* procedure 6

Figure S3: *quantreg* package of R: *crq* procedure 7

Data of the analyzed trials: Mossad (1996)

The data for the Mossad (1996) trial was extracted from survival curves [11], and were published in the Supplement of a previous paper [12].

The table below shows the distribution of cold duration in the two trial arms.

```
> CrossTable( Mossad$Duration, Mossad$Zinc, prop.r ="F", prop.c ="F", prop.t
     ="F", prop.chisq ="F")

Total Observations in Table:  99

                | Mossad$Zinc
Mossad$Duration |          0 |          1 | Row Total |
----------------|------------|------------|-----------|
              1 |          0 |          4 |         4 |
----------------|------------|------------|-----------|
              2 |          4 |          5 |         9 |
----------------|------------|------------|-----------|
              3 |          3 |          6 |         9 |
----------------|------------|------------|-----------|
              4 |          5 |          8 |        13 |
----------------|------------|------------|-----------|
              5 |          2 |          4 |         6 |
----------------|------------|------------|-----------|
              6 |          5 |          6 |        11 |
----------------|------------|------------|-----------|
              7 |          5 |          7 |        12 |
----------------|------------|------------|-----------|
              8 |          5 |          3 |         8 |
----------------|------------|------------|-----------|
              9 |          1 |          3 |         4 |
----------------|------------|------------|-----------|
             10 |          1 |          0 |         1 |
----------------|------------|------------|-----------|
             11 |          2 |          2 |         4 |
----------------|------------|------------|-----------|
             12 |          2 |          0 |         2 |
----------------|------------|------------|-----------|
             13 |          1 |          1 |         2 |
----------------|------------|------------|-----------|
             14 |          2 |          0 |         2 |
----------------|------------|------------|-----------|
             15 |          3 |          0 |         3 |
----------------|------------|------------|-----------|
             16 |          3 |          0 |         3 |
----------------|------------|------------|-----------|
             17 |          2 |          0 |         2 |
----------------|------------|------------|-----------|
             18 |          1 |          0 |         1 |
----------------|------------|------------|-----------|
             19 |          3 |          0 |         3 |
----------------|------------|------------|-----------|
   Column Total |         50 |         49 |        99 |
----------------|------------|------------|-----------|
```

Data of the analyzed trials: three Zinc acetate trials

The data sets of the three RCTs on zinc acetate lozenges [13-15] were provided by the authors of the trials and are shown in the Supplementary files of previous papers [16,18].

The table below shows the distribution of cold duration in the two trial arms.

```
> CrossTable( ZnAcet$Duration, ZnAcet$Zinc, prop.r ="F", prop.c ="F",
prop.t ="F", prop.chisq ="F")

Total Observations in Table:  199
```

	ZnAcet$Zinc		
ZnAcet$Duration	0	1	Row Total
2	3	10	13
3	3	23	26
4	10	21	31
5	10	17	27
6	12	12	24
7	21	8	29
8	14	6	20
9	8	1	9
10	3	1	4
11	2	2	4
12	3	1	4
13	2	0	2
14	2	0	2
15	4	0	4
Column Total	97	102	199

Supplementary Material

STATA *sqreg* procedure for constructing Fig. 2A

```
import delimited "C:\Users\hemila\OneDrive\QTE_Zn\Mossad.csv", encoding(Big5) clear

sqreg duration zinc, quantile(0.05 0.1 0.15 0.2 0.25 0.3 0.35 0.4 0.45 0.5 0.55 0.6 0.65 0.7 0.75 0.8 0.85 0.9 0.95)
        reps(1000)

preserve

gen q = _n*5 in 1/19
list

gen _b_zinc  = .
gen _lb_zinc = .
gen _ub_zinc = .

replace _b_zinc = _b[q05:zinc] in 1
replace _lb_zinc = _b[q05:zinc] - _se[q05:zinc]*invnormal(.975) in 1
replace _ub_zinc = _b[q05:zinc] + _se[q05:zinc]*invnormal(.975) in 1

local i = 2
foreach q of numlist 10(5)95 {
        replace _b_zinc = _b[q`q':zinc] in `i'
      replace _lb_zinc = _b[q`q':zinc] - _se[q`q':zinc]*invnormal(.975) in `i'
      replace _ub_zinc = _b[q`q':zinc] + _se[q`q':zinc]*invnormal(.975) in `i++'
}

keep q _b_* _lb_* _ub_*
keep in 1/19
reshape long _b_ _lb_ _ub_, i(q) j(var) string
set scheme s1color
twoway rarea _lb_ _ub_ q ,  astyle(ci) acolor(%50) || ///
        line _b_ q, lcolor(black) lwidth(0.5)  ///
        yline(0,    lcolor(black) lwidth(0.3) lpattern(dash))  ///
        yline(-4,   lcolor(blue)  lwidth(0.3) lpattern(shortdash))  ///
        subtitle("") ///
        yscale(range(-11.5,1)) ylabel(-8(2)0)   ///
        text( -11 10 "3" -11 16 "4" -11 26 "5" -11 30 "6" -11 40 "7" -11 50 "8" ///
  -11 62 "10" -11 68 "12" -11 74 "14" -11 84 "16"  -11 94 "18", color(red))    ///
  text(-10 10 "Duration in the placebo group (days)", color(red) placement(e)) ///
  text(-5 0 "-4.0 day mean", color(blue) placement(e)) ///
  text(-2 90 "A", size(15)) ///
  by(var, xrescale note(""))  ///
  legend(order(2 "Effect"  1 "95% CI") rows(2) )   ///
  ytitle(Effect on cold duration (days))                    ///
  ylab(,angle(0) format(%7.0gc))                       ///
  xlab(0(20)100) xtitle(Percentile of common cold)  ///
  restore
```

Figure S1: *quantreg* package of R: *rq* procedure

In our paper, we use the STATA procedure *sqreg* because that has more options for modifying the figure, but there are QTE programs also available in the R program set. This below uses steps of 1/1000 to show accurate QTE curve without the 95%CI.

```
MossadRQ <- rq(Duration ~ Zinc, data=Mossad, tau = c(0:950/1000))

plot(MossadRQ)
```

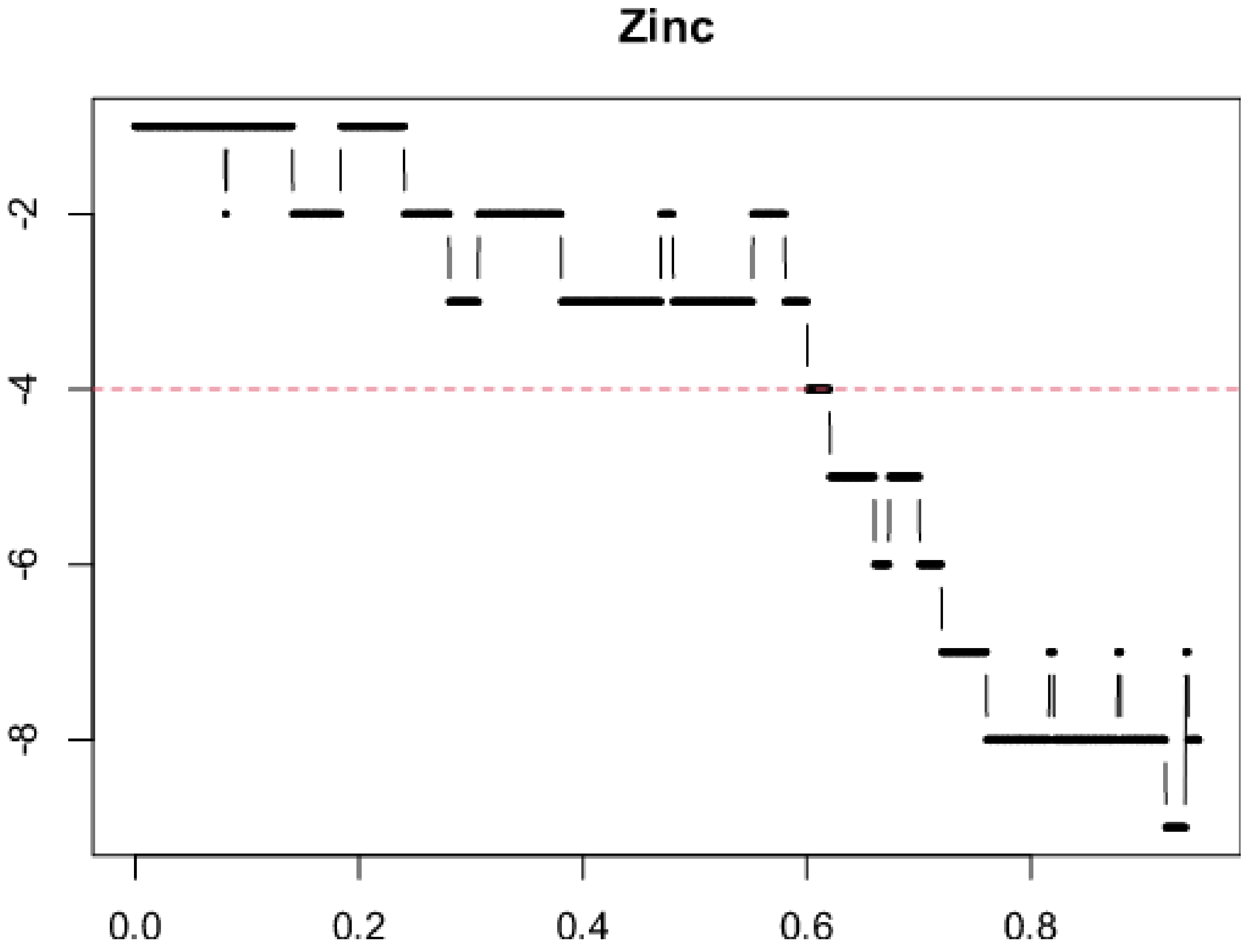

Figure S2: ***qte*** **package of R:** ***ci.qte*** **procedure**

MossadQTE <- ci.qte(Duration ~ Zinc, data=Mossad, probs=seq(0.,0.95, .05), se=T, iters =500)

ggqte(MossadQTE, ybreaks =c(-8, -6, -4, -2, 0, 2, 4), xbreaks =c(0, 0.2, 0.4, 0.6, 0.8, 1.0), setype ="pointwise", ylab ="Effect on cold duration (days)")

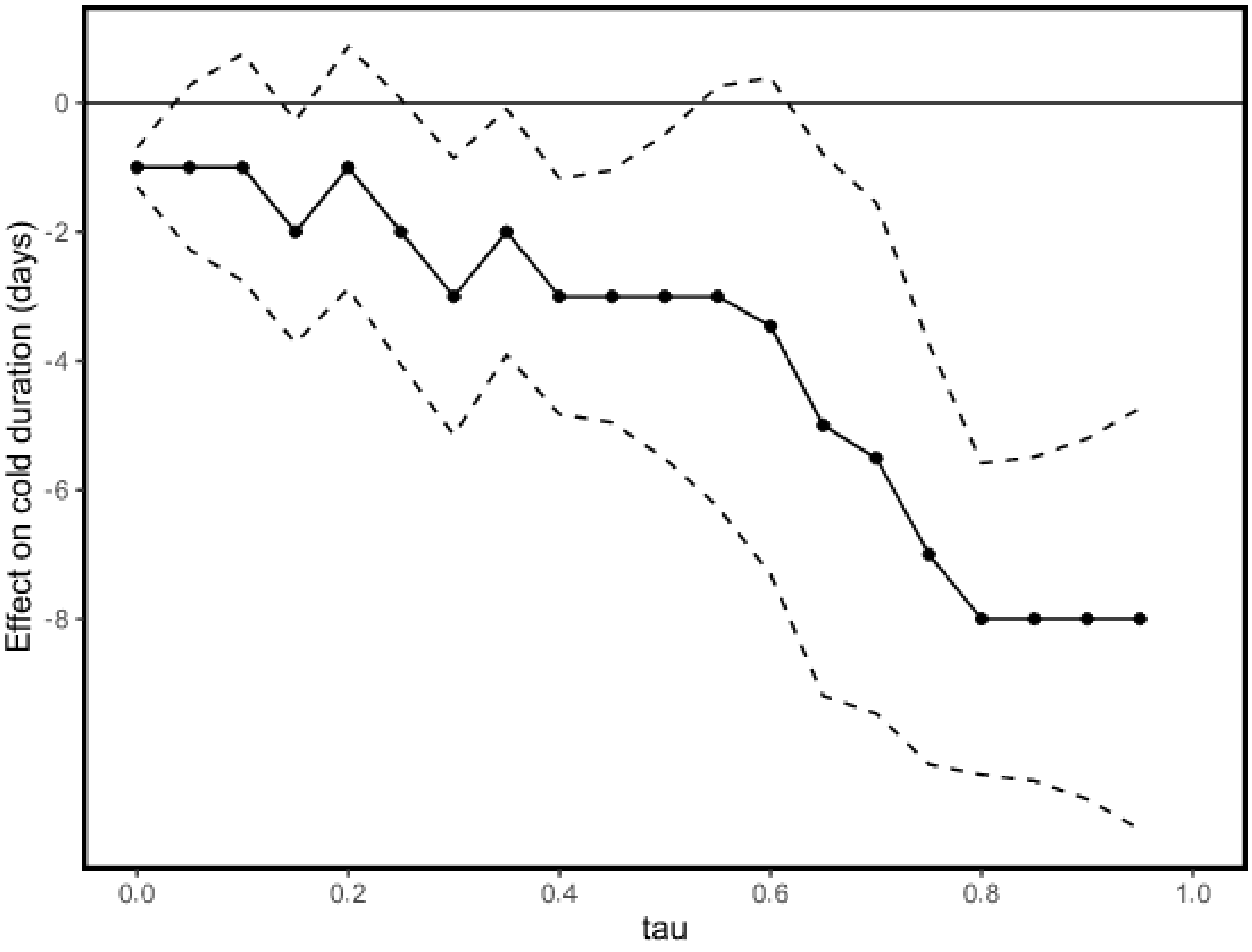

Figure S3: *quantreg* package of R: *crq* procedure

In the Mossad trial there were 8 censored observations (8% of the total): in the placebo group there were two on day 7, one on day 15, one on day 16, and two on day 19; and in the zinc group there was one on day 9 and one on day 11.

The R program *crq* takes into account the censored observations.
The pattern is quite similar to that shown in Fig. 2A which does not take into account censoring.

```
Mossadsurv <- Surv(Mossad$Duration, Mossad$Cured)

MossadCRQ <- crq(Mossadsurv ~ Zinc , data=Mossad, tau = c(1:19/20), method ="PengHuang")

plot(summary(MossadCRQ, 1:19/20, R = 10000), ylim = c(-15, 3),nrow=1,ncol=1, xlab="Quantile of
    common cold duration", ylab ="Effect on cold duration (days)")
```

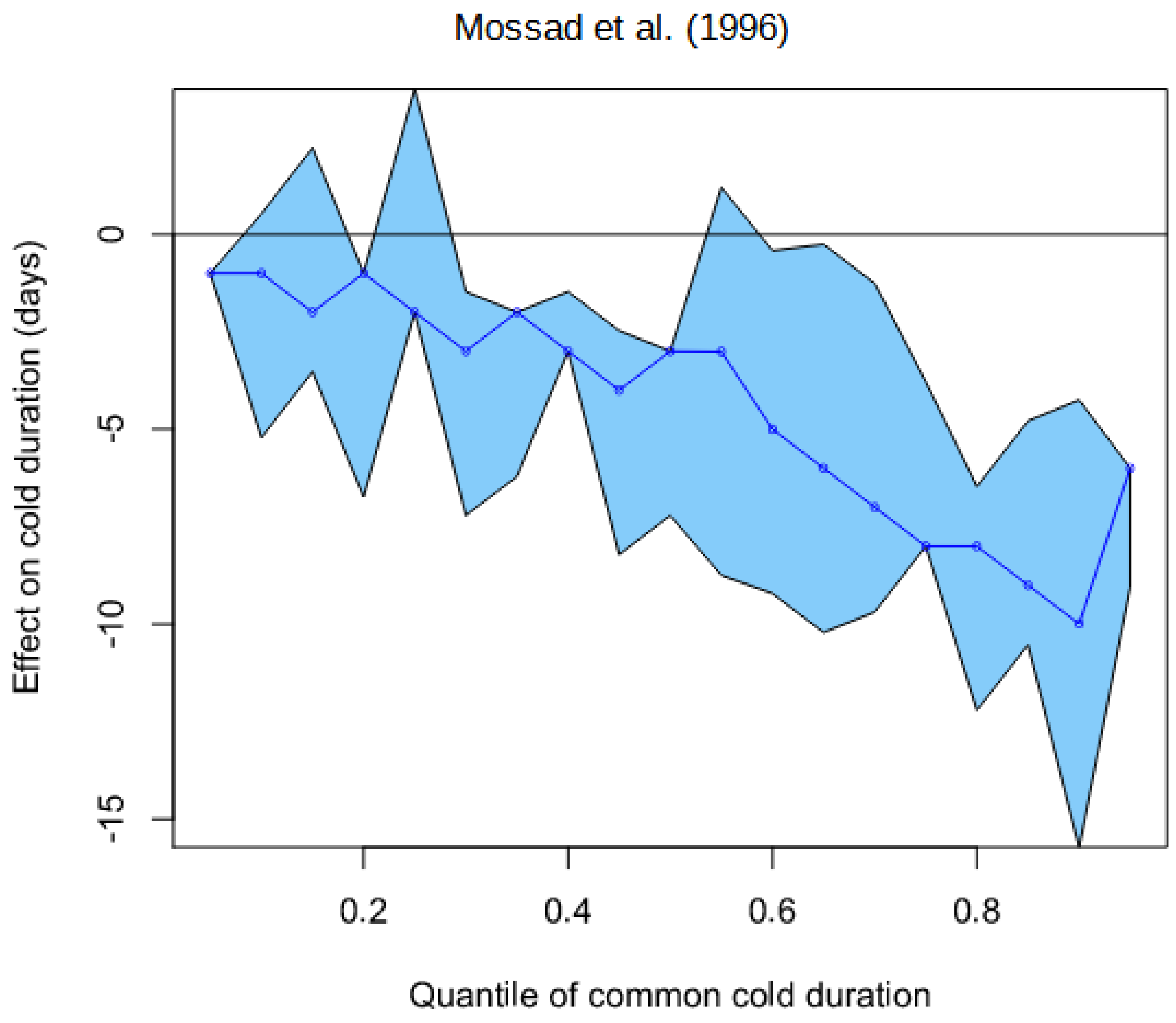

Supplement: Supplementary file 1 [file DataSheet1.PDF]
